# Cod Liver Bread

**Published:** 1874-09

**Authors:** 


### COD LIVER BREAD.

We shall all be dosed to death now certainly; they are beginning to put physic in our bread. Medical journals give assurance that the most satisfactory results have been observed in making Cod Liver Oil Bread, thus: "Mix three tablespoonfuls of the oil with a quantity of warm milk, using as much flour as is necessary to form a consistent dough, which is improved by thorough kneading like other bread. We add the usual allowance of salt, and just a trifle of brown sugar. The bread preserves its moisture longer than the usual home-baked kind, and when toasted is almost undistinguishable from it."

The results will be no doubt satisfactory, very, to closely calculating boarding-house keepers. We should think that one loaf would last an able-bodied man a year. Cod Liver Oil, as a remedy for disease, has been in use in this country less than thirty years, except in limited localities. Since then its employment has been constantly increasing. The strongest testimony we have ever been able to elicit out of the great number of persons who have employed it during that time is that they thought that it did them some good. Three out of four were uncertain whether it did them any good or not. On such slender testimony as this, it may well be accounted a cruelty to advise poor persons to take Cod Liver Oil, and that too in the stages of consumption which the attendant must know are hopeless of cure, and when all the benefit to be possibly derived is a kind of "make-believe" on the patient's mind that something is really doing which may restore to health. Whatever good may have seemed to result in the flat lands and damp climates of Germany and England from taking Cod Liver Oil in consumptive complaints, there is no sure ground for asserting that any benefit was ever derived in the United States from taking this article in consumptive cases, beyond what any other oil would have done. One of the great and ever present complaints of the consumptive is chilliness and consequent great liability to take cold. The oily element does warm the body, but any oil—sweet oil, butter, hog's lard, goose grease, bear's oil—all have the same warming power, and would do just as much good in that direction. Every chemist, every intelligent physiologist knows that oils as food give no strength; they warm, and warm only. Above all things else the consumptive needs strength; it is the ever present want from beginning to end; strength to take exercise, strength to digest the food eaten, strength to wake up a vigorous appetite. Cod Liver Oil always impairs the digestion, because the stomach power required to convert it into vital warmth is so far expended in that direction that there is not enough left to withdraw that nutriment from more solid food, which alone can give any real flesh and strength and weight to the body. Besides, in a great many cases, the use of Cod Liver Oil thrice a day deranges the healthful action of the stomach and the whole alimentary canal. Without the active digestion of wholesome, nourishing food, and the plentiful breathing of a pure out-door air for several hours every day, no actual consumptive ever recovered, and nothing known to this day has ever had any real effect towards modifying the disease, except in proportion as it tended to the promotion of a greater appropriation of out-door air and nourishing food. That Cod Liver Oil in medicinal doses does impair the appetite in consumptive diseases can scarcely be denied.

